# Genetic Targeting of dSAMTOR, A Negative dTORC1 Regulator, during *Drosophila* Aging: A Tissue-Specific Pathology

**DOI:** 10.3390/ijms24119676

**Published:** 2023-06-02

**Authors:** Stamatia A. Katarachia, Sophia P. Markaki, Athanassios D. Velentzas, Dimitrios J. Stravopodis

**Affiliations:** Section of Cell Biology and Biophysics, Department of Biology, School of Science, National and Kapodistrian University of Athens (NKUA), 15701 Athens, Greece; skatarachia@biol.uoa.gr (S.A.K.); smarkak@biol.uoa.gr (S.P.M.); tveletz@biol.uoa.gr (A.D.V.)

**Keywords:** aging, betaine, dBHMT, *Drosophila*, dSAMTOR, dTORC1, kinase, methionine, SAM

## Abstract

mTORC1 regulates mammalian cell metabolism and growth in response to diverse environmental stimuli. Nutrient signals control the localization of mTORC1 onto lysosome surface scaffolds that are critically implicated in its amino acid-dependent activation. Arginine, leucine and S-adenosyl-methionine (SAM) can serve as major mTORC1-signaling activators, with SAM binding to SAMTOR (SAM + TOR), a fundamental SAM sensor, preventing the protein’s (SAMTOR’s) inhibitory action(s) against mTORC1, thereby triggering its (mTORC1) kinase activity. Given the lack of knowledge regarding the role of SAMTOR in invertebrates, we have identified the *Drosophila* SAMTOR homologue (dSAMTOR) in silico and have, herein, genetically targeted it through the utilization of the GAL4/UAS transgenic tool. Survival profiles and negative geotaxis patterns were examined in both control and *dSAMTOR*-downregulated adult flies during aging. One of the two gene-targeted schemes resulted in lethal phenotypes, whereas the other one caused rather moderate pathologies in most tissues. The screening of head-specific kinase activities, via PamGene technology application, unveiled the significant upregulation of several kinases, including the dTORC1 characteristic substrate dp70S6K, in *dSAMTOR*-downregulated flies, thus strongly supporting the inhibitory dSAMTOR action(s) upon the dTORC1/dp70S6K signaling axis in *Drosophila* brain settings. Importantly, genetic targeting of the *Drosophila* BHMT bioinformatics counterpart (dBHMT), an enzyme that catabolizes betaine to produce methionine (the SAM precursor), led to severe compromises in terms of fly longevity, with glia-, motor neuron- and muscle-specific dBHMT downregulations exhibiting the strongest effects. Abnormalities in wing vein architectures were also detected in *dBHMT*-targeted flies, thereby justifying their notably reduced negative geotaxis capacities herein observed mainly in the brain–(mid)gut axis. In vivo adult fly exposure to clinically relevant doses of methionine revealed the mechanistic synergism of decreased dSAMTOR and increased methionine levels in pathogenic longevity, thus rendering (d)SAMTOR an important component in methionine-associated disorders, including homocystinuria(s).

## 1. Introduction

The mechanisms that control organismal growth and its coordination with the availability of nutrients in cellular micro-environments were unknown until a few decades ago. It is now well appreciated that the mechanistic target of rapamycin (mTOR) protein kinase (formerly “mammalian” TOR) represents the major nutrient-sensitive regulator of animal growth [1,2]. The mTOR pathway responds to diverse environmental signals and coordinates cell growth and proliferation [2,3], and its deregulation has been strongly associated with aging and a number of human disorders, such as diabetes, neurodegenerative diseases and cancer [4,5].

mTOR is an evolutionary conserved serine/threonine protein kinase that belongs to the PI3K-related kinase (PIKK) family and is the catalytic core of two large multi-protein complexes named mTORC1 (mTOR complex 1) and mTORC2 (mTOR complex 2) [2,6]. Their function, substrate specificity, upstream inputs, downstream outputs and sensitivity to rapamycin differ notably due to the distinct composition of their accessory proteins [5,6]. mTORC1 is better characterized and reviewed out of the two mTOR complexes. It controls cell growth and metabolism by regulating anabolic and catabolic processes, including protein, lipid and nucleotide synthesis. mTORC1 also blocks autophagy at the post-translational and transcriptional levels in response to various environmental cues, such as growth factors, energy status, oxygen, stress and especially amino acids [3,6,7].

Amino acids, which are not only essential components for protein synthesis but also serve as source of energy and carbon for many metabolic pathways, are also key regulators of mTORC1 [2,4]. Amino acids derive either from lysosome-mediated protein degradation or are exogenously sensed by the GATOR–Rag guanosine triphosphatase (GTPase) pathway, which activates the mTORC1 cascade [8]. Rag GTPases interact with Raptor, a major component of mTORC1, to translocate mTORC1 to the lysosomal membrane, where it is then activated by Rheb. The translocation of mTORC1 requires the interaction of Rag GTPases with Ragulator, which is a complex located on the lysosome surface [9].

mTORC1 is capable of sensing intra-lysosomal and cytosolic amino acid concentrations through distinct mechanisms and sensors [2,10]. Cytosolic arginine and leucine can signal to mTORC1 via different pathways controlled by the GATOR2 and GATOR1 complexes. GATOR1 is a negative regulator of mTORC1 when bound to the lysosome via recruitment by the KICSTOR protein complex, whereas GATOR2 acts as a positive regulator [9]. Leucine and arginine bind to the cytosolic amino acid sensor proteins Sestrin1/2 and CASTOR1, respectively, and dissociate them from GATOR2, which is then activated and triggers the activation of mTORC1 through the inactivation of GATOR1 [11,12]. Remarkably, mTORC1 cannot sense methionine directly, but only indirectly, via a previously uncharacterized protein called SAMTOR. SAMTOR is an S-adenosyl-methionine (SAM) cytoplasmic sensor that interacts with KICSTOR and GATOR1 to release mTORC1 from the lysosomal membrane, thereby acting as a negative regulator of mTORC1. SAM is biochemically produced from methionine, and under methionine sufficiency, SAM binds to SAMTOR, which then dissociates from GATOR1. The disruption of the SAMTOR–GATOR1 complex causes the inactivation of GATOR1, which results in mTORC1 activation [2,7,13].

To investigate the role of SAMTOR in vivo in model biological environments, herein, we have targeted the *Drosophila* homologous gene *dSAMTOR* in diverse fly tissues during aging through the combined employment of GAL4/UAS and RNAi transgenic technology. In addition to *dSAMTOR*, *dBHMT*, the gene controlling the betaine to methionine conversion, was also downregulated, in a tissue-specific manner, throughout the flies’ lifetime. System pathologies derived from gene targeting revealed the in vivo importance of the methionine–SAM axis in cellular integrity, tissue architecture and organ functionality in elderly individuals.

## 2. Results

### 2.1. RNAi-Mediated Downregulation of the Evolutionary Conserved dSAMTOR Protein Causes Either Lethal or Viable Phenotypes

**Figure 1:**
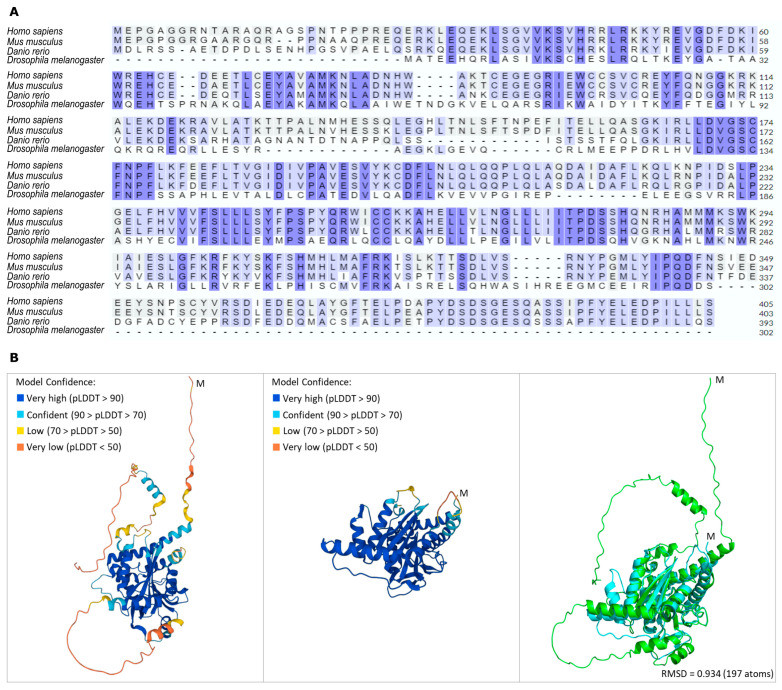
Evolutionary conservation of SAMTOR protein among species. (**A**) Amino acid sequence alignment among human (*Homo sapiens*), mouse (*Mus musculus*), zebrafish (*Danio rerio*) and fly (*Drosophila melanogaster*) SAMTOR proteins, via employment of the Clustal Omega bioinformatics tool. (**B**) Structural alignment of the human (AF-Q1RMZ1-F1) (left panel) and *Drosophila* (AF-Q9W138-F1) (middle panel) SAMTOR proteins derived from the PyMOL (right panel) molecular graphics system [14] (light green: human SAMTOR; light blue: *Drosophila* SAMTOR). The root mean square difference (RMSD) of 0.934 indicates the reliability of the structural alignment.

Cells have a complex system to sense the availability of amino acids, which are crucial activators of mTORC1. SAMTOR, a recently identified protein that serves as an indirect sensor of methionine, has herein been shown to exhibit modular amino acid sequence homology among human (*Homo sapiens*), mouse (*Mus musculus*), zebrafish (*Danio rerio*) and fly (*Drosophila melanogaster*) species, according to the Clustal Omega-mediated alignment (Figure 1A). In accordance with this, the structural alignment of the AlphaFold-derived molecular models has revealed the structural conservation between the human and *Drosophila* SAMTOR proteins, as evidenced by the significant superposition of the coloured, respective (high model confidence), protein segments (Figure 1B).

To investigate the in vivo role of the *Drosophila* homologue dSAMTOR (Figure 1) in the fly’s systemic pathophysiology, we genetically targeted the *dSAMTOR* cognate gene in all organ tissues through the implementation of the appropriate crosses of whole-body or tissue-specific, respectively, GAL4 drivers with two UAS-SAMTOR_RNAi strains, via the combined employment of GAL4/UAS and RNAi transgenic technology. GAL4/UAS is a widely used system in *Drosophila* that allows the selective over-expression of any RNAi under the control of tissue-specific promoters. Briefly, lines expressing the GAL4 yeast transcriptional activator (driver) are crossed with lines carrying the transgene of interest (e.g., RNAi) cloned downstream of the GAL4 binding sites (UAS). In the progeny of a cross between these lines, the RNAi of interest is expressed in the same tissue-specific fashion as the GAL4 activator [15,16,17].

Surprisingly, two distinct phenotypes were obtained, with the first one being typified by embryonic lethality in the majority of *dSAMTOR*-targeted tissues (Table 1) and the second one being characterized by moderate pathologies of age-dependent profiles. It seems that the neuronal system (elav.L-GAL4), glial cell populations (repo-GAL4), motor neurons (D42-GAL4) and muscles (mef2-GAL4) cannot tolerate potent reductions in dSAMTOR protein levels, which are mechanistically associated with systemic lethality (Table 1). In contrast, midgut (NP1-GAL4), eye (ninaE.GMR-GAL4) and wing (bx^MS1096^-GAL4) tissues carrying strong downregulation of *dSAMTOR* gene produced viable offspring (Table 1). As expected, whole-body (Act5C-GAL4) *dSAMTOR* silencing also generated two different phenotypes: a lethal and viable one (Table 1). This indicates the dependence of pathology severity on *dSAMTOR* targeting efficiency, with the “Strong” and “Moderate” strains being named SAMTOR_RNAi^(S)^ and SAMTOR_RNAi^(M)^, respectively (Table 1). In accordance, the moderately reduced expression of the *dSAMTOR* gene in all body tissues (Act5C>SAMTOR_RNAi^(M)^) could not affect (in a statistically significant manner) the survival profiles in male or female transgenic flies compared to control settings (Act5C-GAL4/+) (Figure S1). Taken together, SAMTOR is presented as an evolutionary conserved protein, with the tissue-specific downregulation of the dSAMTOR protein resulting in either systemic mortality or systemic viability, phenotypes that are likely coupled with the targeting efficiency level of the *dSAMTOR* gene.

### 2.2. Differential Contribution of Neuronal Tissue-, Glial Cell-, Motor Neuron- and Muscle Tissue-Specific Moderate Silencing of dSAMTOR Gene to Fly Survival and Kinetic Capacity during Aging

**Figure 2:**
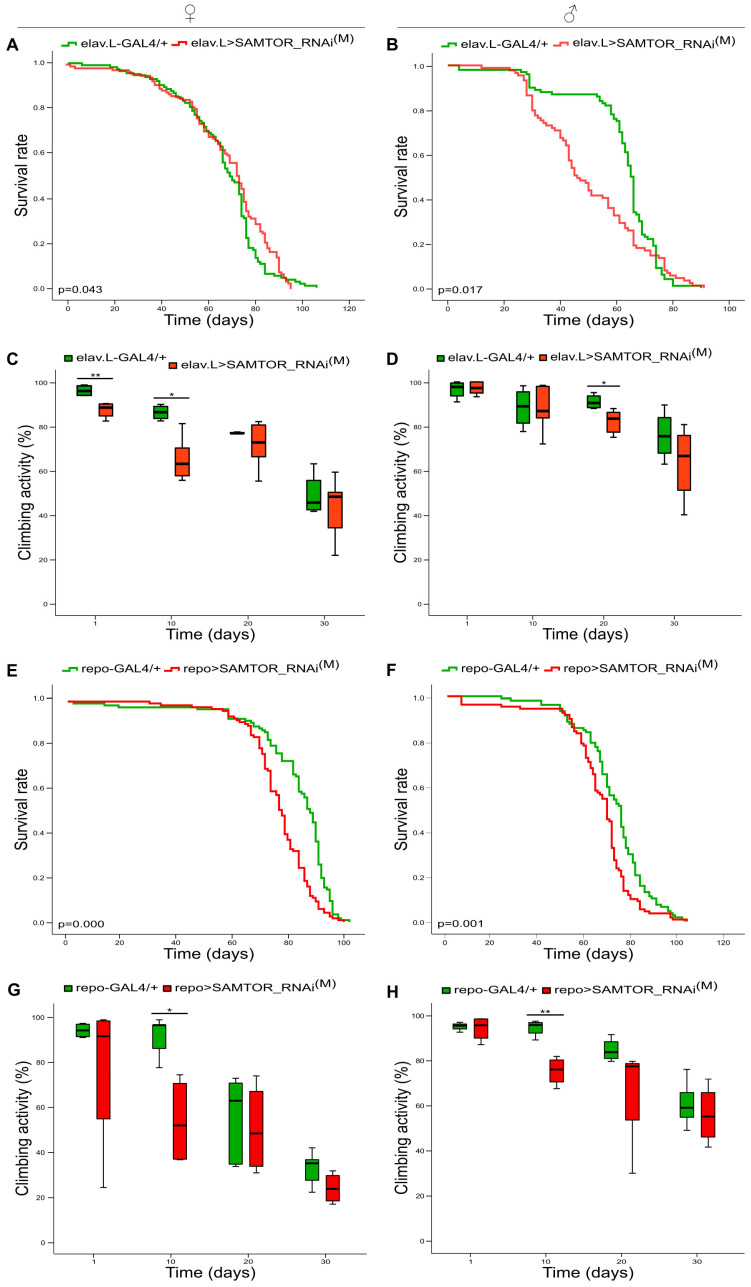
Neuronal tissue- and glial cell-specific mild-proficiency targeting of *dSAMTOR* gene. Curves (**A**,**B**) and bar charts (**C**,**D**) showing the survival rates and (%) climbing capacities, respectively, of female (left panel) and male (right panel) transgenic flies characterized by RNAi-mediated modest targeting of the *dSAMTOR* gene, specifically in neuronal tissues (elav.L>SAMTOR_RNAi^(M)^) (red lines/bars), as compared to control fly populations (elav.L-GAL4/+) (green lines/bars). Curves (**E**,**F**) and bar charts (**G**,**H**) presenting longevity profiles and (%) climbing patterns, respectively, of female (left panel) and male (right panel) flies, with moderate downregulation of the *dSAMTOR* gene, specifically in glial cells (repo>SAMTOR_RNAi^(M)^) (red lines/bars), as compared to control flies (repo-GAL4/+) (green lines/bars). * *p* < 0.05 and ** *p* < 0.01.

Τhe downstream metabolite of methionine S-adenosyl-methionine (SAM) serves as the main methyl group donor for numerous biochemical reactions, including the ones forming mono-amine neurotransmitters, which are molecules used by the nervous system to transmit messages between neurons, or from neurons to other body parts. Hence, to examine if *dSAMTOR*’s moderate downregulation alters the nervous system’s development and function, neuronal cell-specific targeting of *dSAMTOR* gene was carried out via employment of the elav.L-GAL4 genetic driver. Different effects on *Drosophila* longevity were observed after the neuronal lowering of dSAMTOR contents, following sex-dependent profiles. *dSAMTOR*’s moderate suppression significantly increases mortality of male flies (elav.L>SAMTOR_RNAi^(M)^), whereas female flies retain similar viability profiles compared to control conditions (elav.L-GAL4/+) (Figure 2A,Β). The climbing activity (reflecting kinetic potency) of female and male transgenic flies, characterized by RNAi-mediated targeting of the *dSAMTOR* gene, specifically in neuronal tissues, is not notably affected, as compared to control fly populations. Nevertheless, the examined transgenic female and male flies (elav.L>SAMTOR_RNAi^(M)^) show reduced kinetic capacities till the 10th day for the female and the 20th day for the male populations compared to control settings (elav.L-GAL4/+), with all double-targeted flies ultimately recovering by the 30th experimentation day (Figure 2C,D).

In the same context, after the *dSAMTOR* gene’s moderate silencing, specifically in glial cells, life expectancy proves to notably differ from control flies (repo-GAL4/+) in both sexes (repo>SAMTOR_RNAi^(M)^), and is markedly reduced after the 60th day of life (Figure 2E,F). Furthermore, our results demonstrate that kinetic capacities are rather similar between *dSAMTOR*-targeted flies (repo>SAMTOR_RNAi^(M)^) and control populations (repo-GAL4/+), except for the 10th experimentation day, which is characterized by decreased mobility of *dSAMTOR*-downregulated flies for both sexes (repo>SAMTOR_RNAi^(M)^), as compared to control flies (repo-GAL4/+) (Figure 2G,H). Like neuronal targeting, glia-specific (moderate) downregulation of the *dSAMTOR* gene causes kinetic pathologies that can be alleviated during aging (e.g., on the 30th day of life), thereby indicating the critical contribution of dSAMTOR signalling activity to neuronal and glial tissues that control *Drosophila’s* kinetic functionality during their early life. 

**Figure 3:**
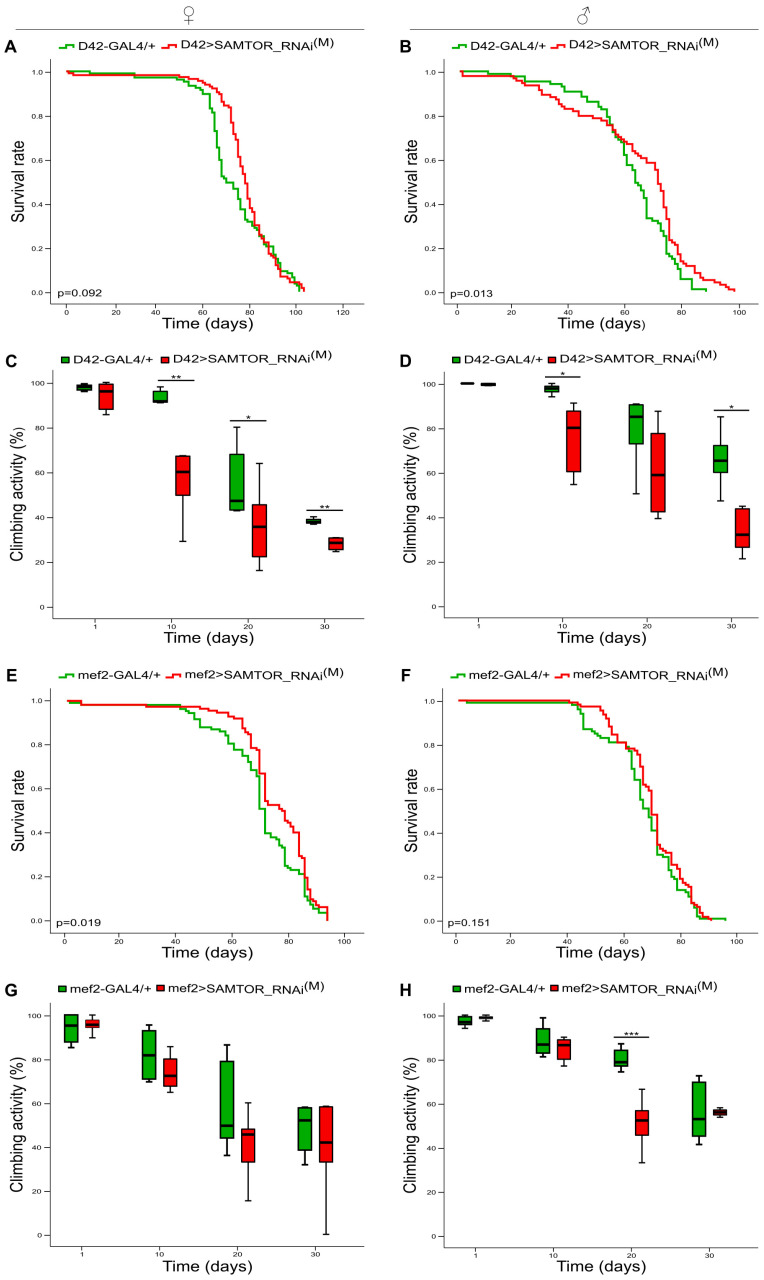
*dSAMTOR* modest suppression in motor neurons and muscles. Lifespan curves (**A**,**B**) and (%) climbing bar charts (**C**,**D**) of female (left panel) and male (right panel) transgenic flies with moderately downregulated *dSAMTOR* gene expression, specifically in motor neurons (D42>SAMTOR_RNAi^(M)^) (red lines/bars), as compared to control fly populations (D42-GAL4/+) (green lines/bars). Lifespan profiles (**E**,**F**) and (%) climbing activity patterns (**G**,**H**) of female (left panel) and male (right panel) flies, with mild downregulation of *dSAMTOR* gene activity, specifically in muscles (mef2>SAMTOR_RNAi^(M)^) (red lines/bars), as compared to control flies (mef2-GAL4/+) (green lines/bars). * *p* < 0.05, ** *p* < 0.01 and *** *p* < 0.001.

To further investigate the role of the dSAMTOR protein in neuro-muscular systemic integrity, the *dSAMTOR* gene was moderately downregulated, specifically either in motor neurons (D42) or in muscles (mef2), via the engagement of the appropriate GAL4 genetic drivers. Different survival profiles were obtained from female and male flies carrying modestly suppressed *dSAMTOR* gene expression, specifically in motor neurons. The longevity of both female and male transgenic flies (D42>SAMTOR_RNAi^(M)^) did not seem to be notably affected (Figure 3A,B), as compared to control populations (D42-GAL4/+), although a tendency of improved viability was observed in female targeted flies. The climbing (negative geotaxis) activities of female and male transgenic flies were rather similar to each other in *dSAMTOR* moderately downregulated settings (D42>SAMTOR_RNAi^(M)^), with the obtained phenotypes featuring significantly compromised mobilities of age-dependent pathologies that could not be rescued on the 30th day of life (Figure 3C,D).

Moderate silencing of *dSAMTOR* gene expression, specifically in muscles, does not seem to alter either males’ longevity (Figure 3F) or the kinetic capacities of both sexes, except for on day 20 in males (significantly reduced) (mef2>SAMTOR_RNAi^(M)^), compared to control transgenic animals (mef2-GAL4/+) (Figure 3G,H). Only transgenic females bearing modest downregulation of dSAMTOR protein levels (mef2>SAMTOR_RNAi^(M)^) showed increased viability (in a statistically significant manner) compared to control fly populations (mef2-GAL4/+) (Figure 3E). Altogether, it seems that moderate reductions in dSAMTOR protein levels in motor neurons or muscles may benefit the longevity of female flies but can harm climbing (locomotor) functions in age-dependent and tissue-specific ways.

### 2.3. dSAMTOR Midgut-Specific Suppression Alters Drosophila’s Longevity

**Figure 4:**
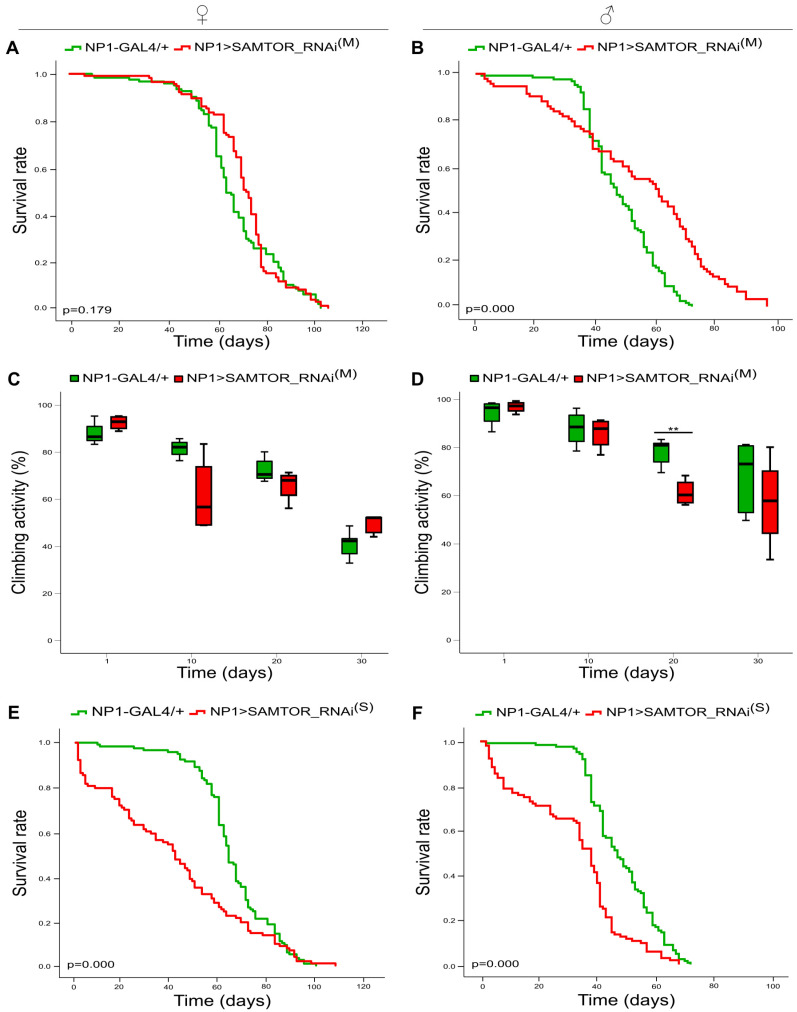
Differential downregulation of *dSAMTOR* gene expression in midgut tissues. Lifespan profiles (**A**,**B**) and bar charts showing (%) kinetic activities (**C**,**D**) of female (left panel) and male (right panel) transgenic flies with moderate *dSAMTOR* silencing capacity, specifically in midgut tissues (NP1>SAMTOR_RNAi^(M)^) (red lines/bars), as compared to control fly populations (NP1-GAL4/+) (green lines/bars). Curves (**E**,**F**) presenting the survival rates of female (left panel) and male (right panel) transgenic flies with strong RNAi-mediated targeting of *dSAMTOR* gene, specifically in midgut tissues (NP1>SAMTOR_RNAi^(S)^) (red lines/bars), as compared to control flies (NP1-GAL4/+) (green lines/bars). SAMTOR_RNAi^(M)^: RNAi strain with moderate (M) *dSAMTOR* silencing capacity. SAMTOR_RNAi^(S)^: RNAi strain with strong (S) *dSAMTOR* silencing potency. ** *p* < 0.01.

*D. melanogaster’s* viability after moderate *dSAMTOR* gene silencing, specifically in midgut tissues, is affected in a sex-dependent manner. Male transgenic flies (NP1>SAMTOR_RNAi^(M)^) show improved viability after day 40 of their life in a statistically significant manner compared to control groups (NP1-GAL4/+) (Figure 4B). In contrast, the lifespan profiles of moderately *dSAMTOR*-targeted female flies do not differ significantly from control animals (Figure 4A). Nevertheless, the climbing capacities of both female and male transgenic flies with modestly *dSAMTOR*-downregulated activity levels (NP1>SAMTOR_RNAi^(M)^) seem to statistically resemble those of control populations (NP1-GAL4/+), although a notable reduction can be observed on the 20th day of males’ life (Figure 4C,D). Remarkably, strong RNAi-mediated suppression of *dSAMTOR* gene expression, specifically in midgut tissues, causes strikingly reduced longevity in transgenic flies of both sexes (NP1>SAMTOR_RNAi^(S)^) compared to control settings (NP1-GAL4/+) (Figure 4E,F). The climbing activity experiments could not be implemented in a reliable fashion due to the increased mortality of transgenic flies. Taken together, it is the different efficiency of the *dSAMTOR* gene targeting and/or downregulation process that directs the two distinct pathologies in midgut tissues detected herein. The more severe phenotype (highly compromised longevity) markedly tolerates embryonic lethality, in contrast to other tissue type-specific (e.g., nervous system and muscles) strong silencing schemes of *dSAMTOR* gene expression, as described in Table 1. Since the midgut serves as the first nutritional entrance and food management hub in flies’ bodies, it has to resist any amino acid-dependent imbalance in basic metabolism, biosynthesis and signalling (likely induced by a lack of dSAMTOR), thereby providing strong support for the nourishment of other organic systems, such as the brain, glia, motor neurons and muscles.

### 2.4. Functional Kinome Profiling of Mildly dSAMTOR-Downregulated Transgenic Flies, Specifically in Neuronal Tissues

Global profiling of the functional (active/ated) kinome is a recently developed, novel, versatile, multi-faceted and powerful molecular platform that offers the opportunity to screen and map several hundreds of protein kinase activities in a given biological specimen in vitro. Kinase-mediated phosphorylation plays a critical regulatory role in many cellular processes, and protein kinases are decisively involved in multiple and diverse cellular functions, including, among others, cell proliferation, differentiation and migration. Kinases represent a special and unique class of drug targets, capable of opening new windows for the development of novel targeted therapies for various diseases. The PamGene platform is a well-established micro-array technology platform for simultaneous multiplex kinase-activity profiling for several hundred (>430) protein kinases in complex biological samples, and, thus, can help in bridging the gap between animals or in vitro models and human diseases.

**Figure 5:**
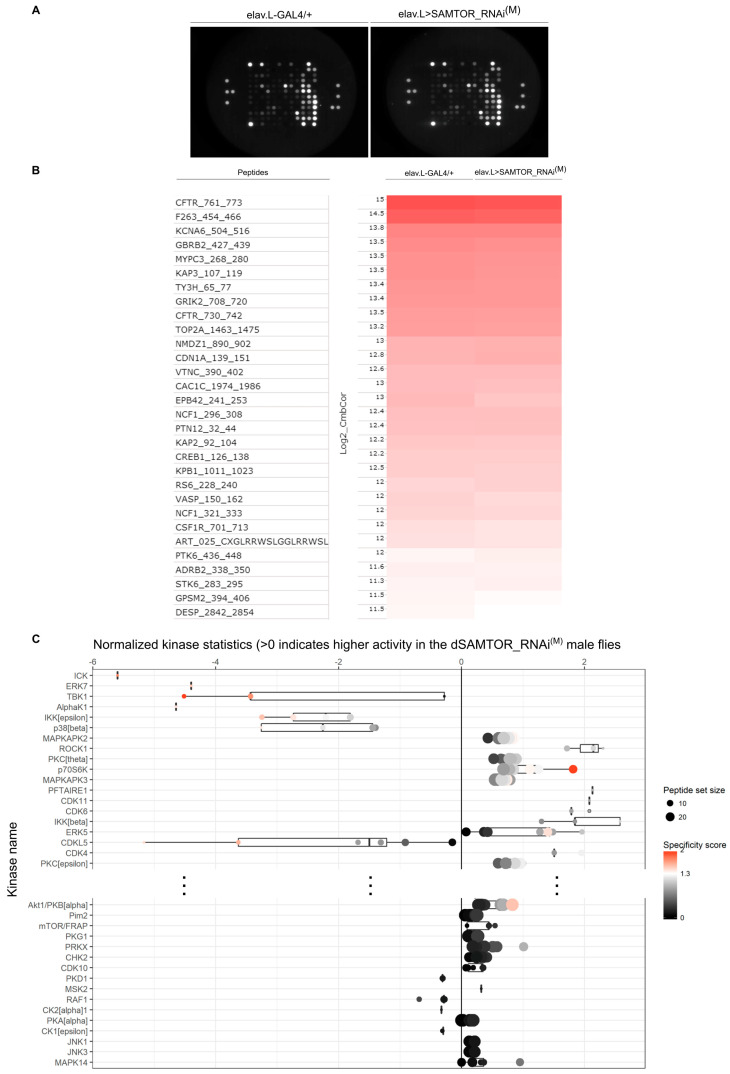
Active kinome analysis of *dSAMTOR*-targeted *D. melanogaster* brains. (**A**) CCD images of PamChip arrays. Differential substrate phosphorylation comparing transgenic flies characterized by RNAi-mediated (modestly efficiency) targeting of the *dSAMTOR* gene, specifically in neuronal tissues (elav.L>SAMTOR_RNAi^(M)^) and control flies (elav.L-GAL4/+), showing representative Ser/Thr protein kinase activities. (**B**) Heatmaps visualizing the first thirty Ser/Thr-peptide phosphorylation intensities (log2) of control and *dSAMTOR*-downregulated flies, specifically in fly brains (neuronal system). (**C**) Median final score plots of the Ser/Thr protein kinase activity profiling in *dSAMTOR*-targeted fly brain (neuronal system) setting. Normalized kinase activity statistics is a mathematical-based algorithm indicating the estimated relative kinase activity, while the specificity score reflects the reliability and accuracy of the prediction. In particular, the x-axis indicates the values for the normalized kinase activity statistics (e.g., negative value (s < 0)  =  “the activity of the corresponding Ser/Thr kinase is decreased”). The specificity score (Qsp) is indicated by the colour of the points. Qsp logarithmic values > 1.3 (white (Qsp = 1.3) to red colour (Qsp ≥ 2)) were considered as statistically relevant.

Hence, in our study, we used the PamGene Ser/Thr Kinase (STK) chips, which are composed of a grid of reporter peptides derived from the literature or computational predictions that can be phosphorylated by Ser/Thr protein kinases present in total-protein lysates of control (elav.L-GAL4/+) and moderately *dSAMTOR*-downregulated (elav.L>SAMTOR_RNAi^(M)^), manually dissected *Drosophila* heads. Phosphorylated peptides were recognized by phospho-specific and FITC-labeled antibodies, while signal detection was performed in multiple cycles at different exposure times and captured using a high-resolution CCD camera. In mildly *dSAMTOR*-targeted transgenic flies, specifically in neuronal tissues (elav.L>SAMTOR_RNAi^(M)^), there is a differential substrate phosphorylation pattern detected compared to control populations (elav.L-GAL4/+), as shown in the captured CCD images of the PamChip micro-arrays (Figure 5A). Peptides with significant differences in signal intensities are visualized after normalization in log-transformed heatmaps (Figure 5B), which demonstrate the degree of phosphorylation for each peptide per genetic condition (control or *dSAMTOR* modest suppression). Signals are sorted from high to low intensity/phosphorylation for control animals and flies with moderately silenced *dSAMTOR* expression. Figure 5C describes the median final score plots, indicating putative Ser/Thr protein kinases ranked by their specificity score. Importantly, p70S6K (a bona fide substrate of activated mTOR kinase) is clustered in the top-ranked (Ser/Thr) functional (active/ated) kinases in the *dSAMTOR* mildly downregulated fly heads, as compared to control settings. Of note, the x-axis indicates the values for normalized kinase activity statistics (e.g., positive value = “activity of the corresponding Ser/Thr protein kinase is increased in modestly *dSAMTOR*-silenced transgenic flies”). Altogether, an active/ated kinome-wide mapping of *Drosophila* heads in wild-type and mutant environments reveals the negative role of the dSAMTOR determinant in the dTOR-dp70S6K signalling axis in vivo. The contribution of other functionally upregulated (e.g., ERK5, ROCK1 and IKKβ) or downregulated (e.g., TBK1 and IKKε) Ser/Thr protein kinases to the obtained pathologies in *dSAMTOR* modestly suppressed settings (Figure 5C) is a novel and interesting mechanistic issue that necessitates further exploration.

### 2.5. Strong Downregulation of dSAMTOR Gene Expression Causes Eye and Wing Dysmorphic Phenotypes in Drosophila

**Figure 6:**
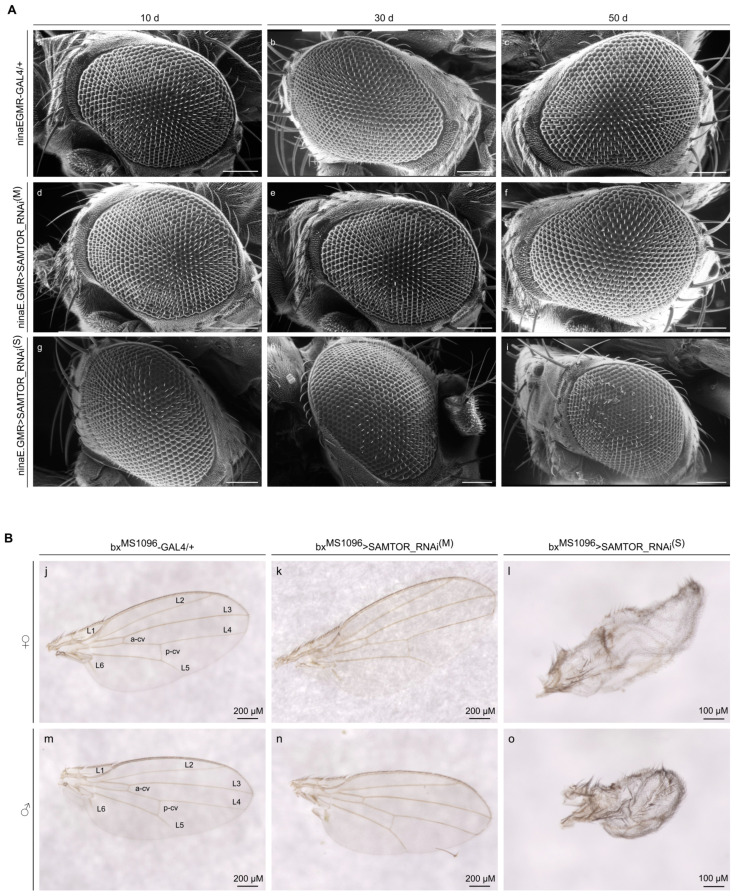
Different levels of *dSAMTOR* gene downregulation result in distinct patterns of eye and wing pathology in *Drosophila*. (**A**) Scanning electron microscopy (SEM) images of compound eyes derived from male transgenic flies at the age of 10, 30 and 50 days, with moderate (M) (ninaE.GMR>SAMTOR_RNAi^(M)^) (**d**–**f**) and strong (S) (ninaE.GMR>SAMTOR_RNAi^(S)^) (**g**–**i**), respectively, RNAi-mediated targeting of the *dSAMTOR* gene, as compared to control settings (ninaE.GMR-GAL4/+) (**a**–**c**). Scale bars: 100 μm. (**B**) Wing images of female mutant flies (top panel) carrying moderate (M) (bx^MS1096^>SAMTOR_RNAi^(M)^) (**k**) and strong (S) (bx^MS1096^>SAMTOR_RNAi^(S)^) (**l**), respectively, *dSAMTOR* gene silencing, as compared to control fly populations (bx^MS1096^-GAL4/+) (**j**). Wing images of male mutant flies (bottom panel) with moderate (M) (bx^MS1096^>SAMTOR_RNAi^(M)^) (**n**) and strong (S) (bx^MS1096^>SAMTOR_RNAi^(S)^) (**o**) *dSAMTOR* gene downregulation, as compared to control fly wings (bx^MS1096^-GAL4/+) (**m**). Scale bars: (**j**,**k**,**m**,**n**): 200 µm; (**l**,**o**): 100 μm. SAMTOR_RNAi^(M)^: RNAi strain with moderate (M) silencing capacity. SAMTOR_RNAi^(S)^: RNAi strain with strong (S) silencing potency. Naming of normal (control) wing veins is indicated (L1–L6, a-cv and p-cv) in (**j**,**m**).

To reveal the role of the dSAMTOR protein in eye and wing morphogenesis during *Drosophila* aging, herein, we targeted *dSAMTOR* gene expression, specifically in the compound eye and wing disc, respectively, via crossing of the appropriate GAL4-genetic drivers with the UAS-SAMTOR_RNAi^(S)^ or UAS-SAMTOR_RNAi^(M)^ transgenic strains. Our results show that moderate downregulation of the *dSAMTOR* gene in the eye disc (ninaE.GMR>SAMTOR_RNAi^(M)^) (Figure 6A(d–f)) does not detectably affect the normal structure and organization of the compound eye during aging, whereas strong *dSAMTOR* gene silencing (ninaE.GMR>SAMTOR_RNAi^(S)^) (Figure 6A(g–i)) significantly alters eyes’ morphology and architecture, with cilia loss following an age-dependent dysmorphic pattern compared to control conditions (ninaE.GMR-GAL4/+) (Figure 6A(a–c)).

Control (bx^MS1096^-GAL4/+) fly wings are characterized by the typical morphogenetic structure being comprised of five main veins (L1-L5), a posterior cross-vein (P-CV) and an anterior cross-vein (A-CV), both in female (Figure 6B(j)) and male (Figure 6B(m)) flies. Moderately suppressed *dSAMTOR* gene activity in female (Figure 6B(k)) and male (Figure 6B(n)) flies (bx^MS1096^>SAMTOR_RNAi^(M)^) seems to be unable to induce any detectable alteration in wing organization and architecture. However, strongly reduced levels of the dSAMTOR protein, specifically in the wing disc, cause severe pathologies for both sexes (bx^MS1096^>SAMTOR_RNAi^(S)^), since wings’ structural architecture is totally disrupted, carrying highly dysmorphic features in both transgenic female (Figure 6B(l)) and male (Figure 6B(o)) flies. Like the midgut (Figure 4), eye and wing disc tissues can also resist embryonic lethality with a lack of dSAMTOR (Table 1), thus indicating their ability to compensate for SAM-dSAMTOR-dTOR axis signalling disruption to obviate the system’s mortality.

### 2.6. Sensing and Metabolism of Methionine Are Evolutionary Conserved Pathways

**Figure 7:**
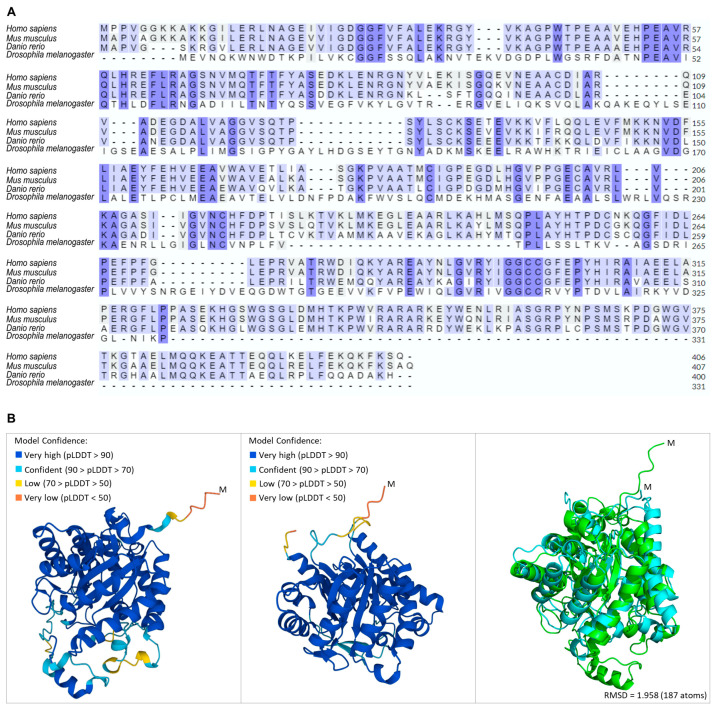
(**A**) Evolutionary conservation of BHMT enzyme among species. Amino acid sequence alignment among human (*Homo sapiens*), mouse (*Mus musculus*), zebrafish (*Danio rerio*) and fly (*Drosophila melanogaster*) BHMT proteins, via employment of the Clustal Omega bioinformatics tool. (**B**) Structural alignment of the human (AF-Q93088-F1) (left panel) and *Drosophila* (AF-Q9VJ31-F1) (middle panel) BHMT proteins as derived from the PyMOL (right panel) molecular graphics system [14] (light green: human BHMT; light blue: *Drosophila* BHMT).

Methionine, an essential amino acid required for organisms’ normal growth and development, is converted to S-adenosyl-methionine (SAM), a major methyl donor in cells, by an enzyme called methionine S-adenosyl-transferase (MAT). SAM binds to SAMTOR, de-repressing mTORC1 signalling activities. Methionine is supplied by nutrition or is endogenously produced from homocysteine via methionine synthase (MTR) or betaine homocysteine methyl-transferase (BHMT) involvement (Figure S2). Through engagement of the Clustal Omega bioinformatics tool, we demonstrate that the BHMT protein is evolutionary conserved in its amino acid sequence among human (*Homo sapiens*), mouse (*Mus musculus*), zebrafish (*Danio rerio*) and fly (*Drosophila melanogaster*) species (Figure 7A). In accordance with this, the structural alignment of the AlphaFold-derived molecular models has unveiled the structural conservation between the human and *Drosophila* BHMT proteins, as shown by the notable superposition of the coloured, respective, protein segments (Figure 7B), thereby providing a new and powerful genetic frame for the mechanistic association of *dBHMT* gene targeting with age-dependent pathologies in *Drosophila* tissues and organs.

### 2.7. Methionine and Betaine Administration Induce Reduced Viability in Transgenic Flies with Moderately Suppressed dSAMTOR Gene Expression, Specifically in Neuronal Tissues

**Figure 8:**
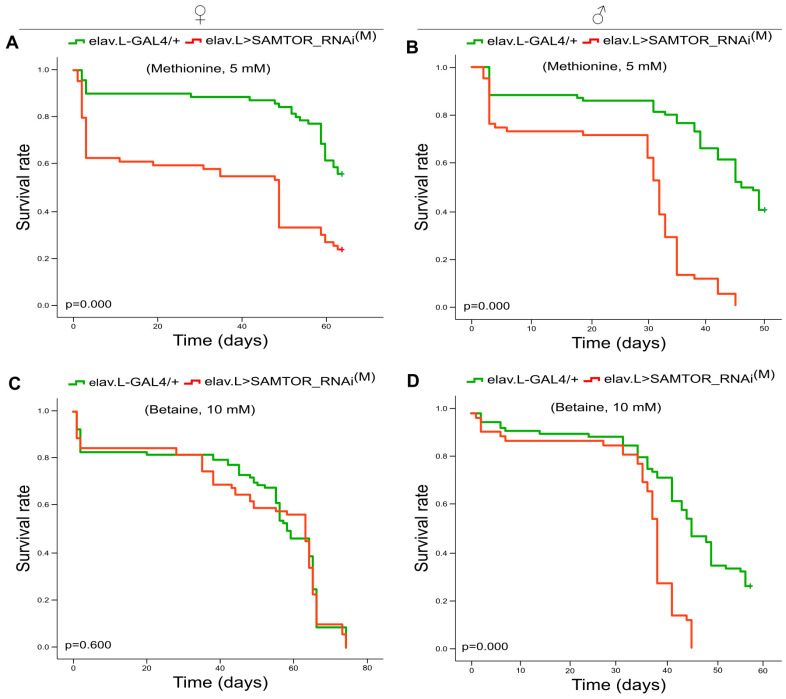
Methionine and betaine treatment of *dSAMTOR* mildly targeted flies, specifically in neuronal tissues, resulted in reduced viability. (**A**,**B**) Survival curves of female (left panel) and male (right panel) *dSAMTOR* moderately downregulated flies, specifically in neuronal tissues (elav.L>SAMTOR_RNAi^(M)^), in the presence of methionine (5 mM) (supplemented into the food) for ~60 consecutive days (red lines), as compared to methionine-exposed control (elav.L-GAL4/+) populations (green lines). (**C**,**D**) Survival rates of female (left panel) and male (right panel) *dSAMTOR* modestly targeted flies, specifically in neuronal tissues (elav.L>SAMTOR_RNAi^(M)^), after betaine administration (10 mM) (in the food) for ~70 consecutive days (red lines), as compared to control (elav.L-GAL4/+) flies treated with the same dose of betaine (green lines).

Besides the RNAi-mediated genetic downregulation of the dSAMTOR protein, we also attempted the exogenous administration (in the food) of the methionine or betaine (trimethylglycine) amino acids and subsequent monitoring of transgenic flies’ viability in modest *dSAMTOR* gene silencing settings, specifically in neuronal tissues. After their exposure to methionine (5 mM; administered in the food) for up to 60 consecutive days, female and male flies, characterized by moderately suppressed expression of the *dSAMTOR* gene (elav.L>SAMTOR_RNAi^(M)^) (specifically in the neuronal system), presented remarkably increased mortality compared to control animals (elav.L-GAL4/+) (Figure 8A,B).

Likewise, the supplementation of food with betaine (10 mM) resulted in a prominent reduction in longevity in male transgenic flies (elav.L>SAMTOR_RNAi^(M)^) (Figure 8D) compared to control populations (elav.L-GAL4/+), although transgenic female lifespan profiles did not seem to differ (in a statistically significant manner) from control cross-derived ones (Figure 8C). The synergism of methionine food enrichment with the neuronal-specific mild downregulation of dSAMTOR protein contents in lifespan decrease indicates the in vivo ability of our genetic scheme developed herein to, indeed, target and suppress *dSAMTOR* gene expression at detectable and productive, albeit moderate, levels. Similarly, betaine can synergize with neural system-specific dSAMTOR modest downregulation in diminishing lifespan, exclusively in male, but not female, transgenic flies. This sex-dependent response may be mechanistically related to the absence (or compromise) of the genomic imprinting process (e.g., betaine metabolism) in *Drosophila* adult females, as previously reported [18]. The presumable duplication of allele expression dose in females likely attenuates the RNAi machinery efficacy, thereby justifying the lack of pathology in transgenic female flies in betaine-enriched environments.

### 2.8. Lack of dBHMT Gene Expression, Specifically in Neuronal, Glial, Motor Neuron, Muscle and Midgut Tissues, Shortens Drosophila’s Longevity

**Figure 9:**
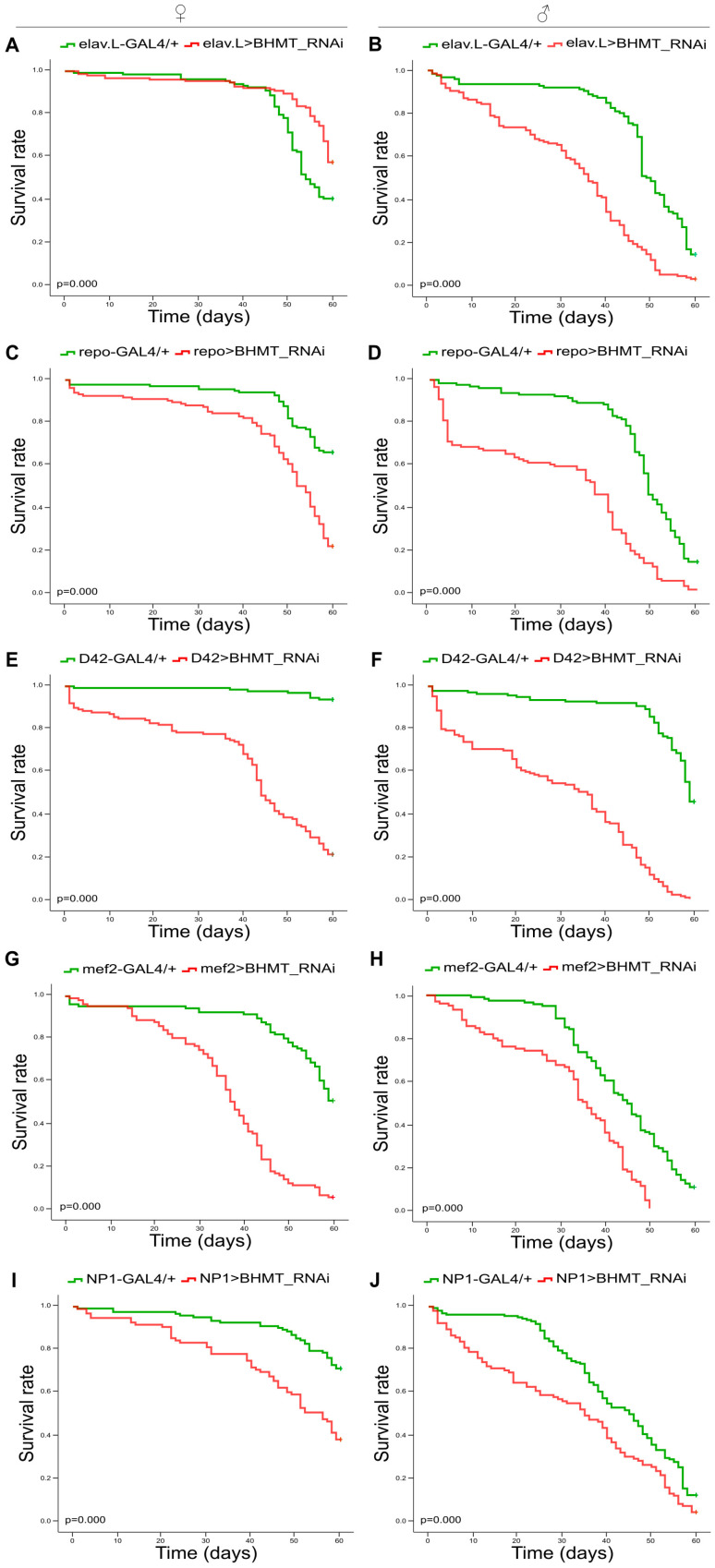
*dBHMT* gene suppression reduces *Drosophila*’s viability in diverse type of tissues. Lifespan profiles of *Drosophila* female (left panels) and male (right panels) transgenic flies characterized by RNAi-mediated and tissue-specific targeting of the *dBHMT* gene in: (**A**,**B**) neuronal tissues (elav.L>BHMT_RNAi), (**C**,**D**) glial cells (repo>BHMT_RNAi), (**E**,**F**) motor neurons (D42>BHMT_RNAi), (**G**,**H**) muscles (mef2>BHMT_RNAi) and (**I**,**J**) midgut tissues (NP1>BHMT_RNAi).

RNAi-mediated *dBHMT* gene silencing, specifically in neuronal tissues, significantly increases the mortality of male flies (elav.L>BHMT_RNAi) compared to control settings (elav.L-GAL4/+) (Figure 9B), whereas *dBHMT*-targeted female flies have elevated viability in older ages (after day 45) (Figure 9A). Most importantly, the suppression of *dBHMT* gene expression, specifically in glial cells (repo>BHMT_RNAi) (Figure 9C,D), motor neurons (D42>BHMT_RNAi) (Figure 9E,F), muscle tissues (mef2>BHMT_RNAi) (Figure 9G,H) and the midgut (NP1>BHMT_RNAi) (Figure 9I,J), leads to remarkably reduced longevities of both female and male flies compared to control populations (repo-GAL4/+), (D42-GAL4/+), (mef2-GAL4/+) and (NP1-GAL4/+), respectively. Taken together, it seems that dBHMT enzymatic activity plays an indispensable role in diverse tissue- and organ-dependent survival programs in *Drosophila* during aging.

### 2.9. Differential Contribution of Neuronal, Glial, Motor Neuron, Muscle and Midgut Tissue-Specific Silencing of dBHMT Gene Expression to Drosophila Locomotion during Aging

**Figure 10:**
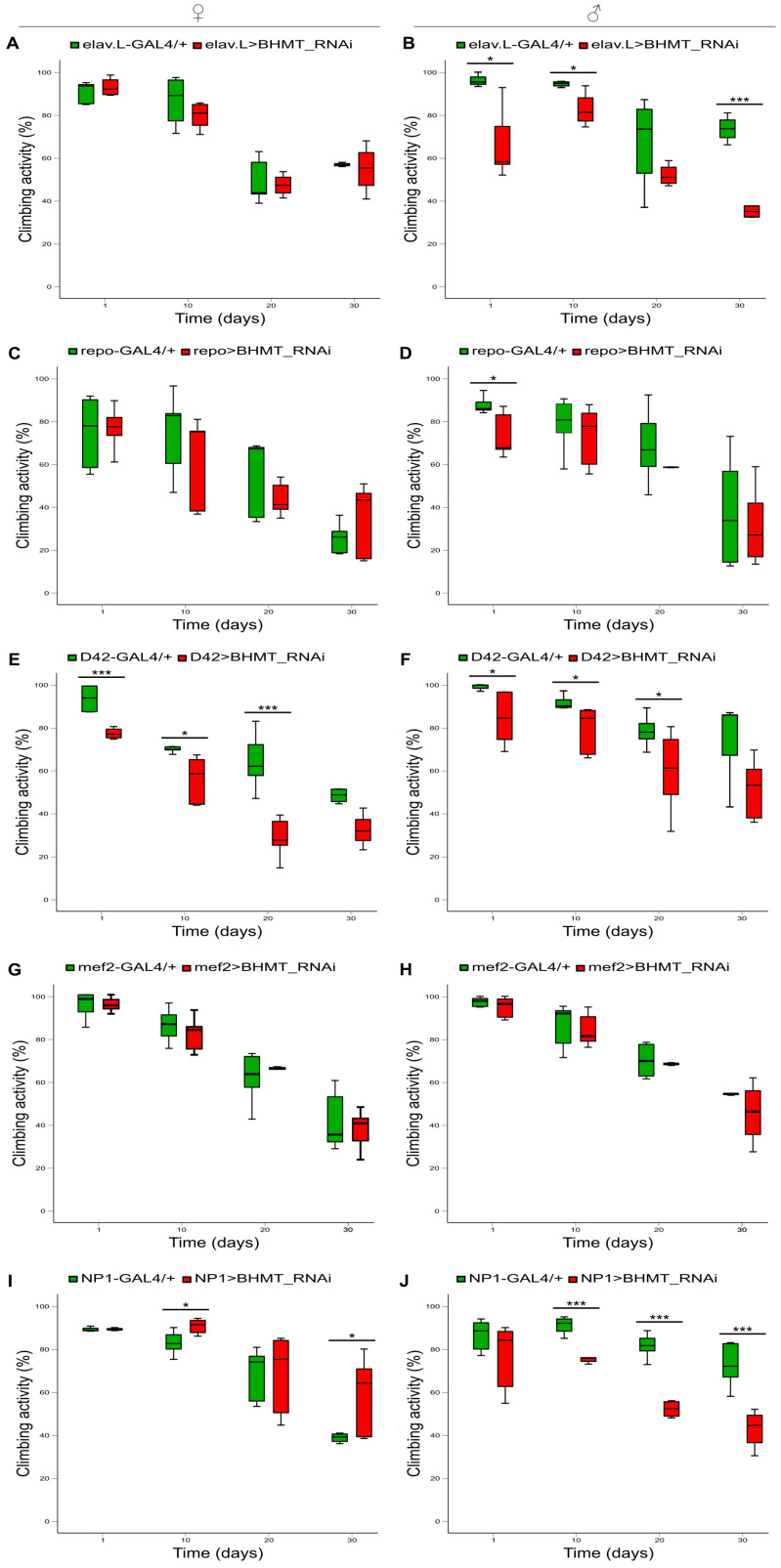
Downregulation of *dΒHΜΤ* gene expression differentially impairs climbing capacity during *Drosophila* aging, revealing tissue-specific signatures. Climbing activity (negative geotaxis) patterns of female (left panels) and male (right panels) transgenic flies characterized by tissue-specific *dΒHΜΤ* gene targeting in: (**A**,**B**) neuronal tissues (elav.L>BHMT_RNAi), (**C**,**D**) glial cells (repo>BHMT_RNAi), (**E**,**F**) motor neurons (D42>BHMT_RNAi), (**G**,**H**) muscles (mef2>BHMT_RNAi) and (**I**,**J**) midgut tissues (NP1>BHMT_RNAi). * *p* < 0.05 and *** *p* < 0.001.

A lack of dBHMT protein synthesis, specifically in neuronal tissues, causes sex-dependent kinetic pathologies, with climbing (negative geotaxis) activities being reduced in male populations (elav.L>BHMT_RNAi) (Figure 10B), but remaining unaffected in female flies (Figure 10A) compared to control groups (elav.L-GAL4/+). Locomotor functions of female and male *dBHMT*-downregulated flies, specifically in glial cells (repo>BHMT_RNAi) (Figure 10C,D) or muscle tissues (mef2>BHMT_RNAi) (Figure 10G,H), do not seem to differ (in a statistically significant manner) from control genetic environments (repo-GAL4/+ and mef2-GAL4/+, respectively). Remarkably, motor neuron-specific silencing of the *dBHMT* gene (D42>BHMT_RNAi) causes a notable reduction in motility for both *Drosophila* sexes during aging (Figure 10E,F). Pathogenic climbing activity profiles are also obtained in response to midgut-specific downregulation of *dBHMT* gene expression (NP1>BHMT_RNAi), revealing sex-dependent kinetic pathologies. Interestingly, *dBHMT*-targeted female flies, specifically in their midgut tissues (NP1>BHMT_RNAi), have elevated kinetic capacity (Figure 10I), whereas transgenic males’ locomotor performance is shown to be prominently deteriorated during aging compared to control populations (NP1-GAL4/+) (Figure 10J). Altogether, it seems that different tissues and organs can unequally contribute to kinetic pathologies derived from *dBHMT* genetic targeting and silencing.

### 2.10. dBHMT Gene Downregulation Critically Affects Drosophila Eye and Wing Development

**Figure 11:**
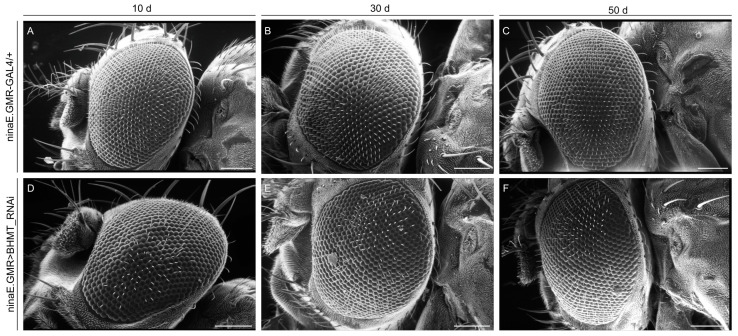
*dBHMT* gene downregulation causes eye dysmorphia in *Drosophila*: an age-dependent pathology. Scanning electron microscopy (SEM) images of compound eyes derived from control (ninaE.GMR-GAL4/+) (**A**–**C**) and *dBHMT*-targeted (ninaE.GMR>BHMT_RNAi) (**D**–**F**), specifically in the eye, male transgenic flies at the age of 10, 30 and 50 days. Scale bars: 100 μm.

The suppression of *dBHMT* gene expression in *Drosophila’s* compound eye disc tissues (ninaE.GMR>BHMT_RNAi) proves to markedly disrupt normal eye cilia formation and distribution during aging, since from the 10th day of life, there is a progressive loss of eye cilia observed, while on day 50, an absence of cilia is detected to a comparatively greater extent (Figure 11D–F) in *dBHMT*-targeted (male) flies compared to control settings (ninaE.GMR-GAL4/+) (Figure 11A–C).

**Figure 12:**
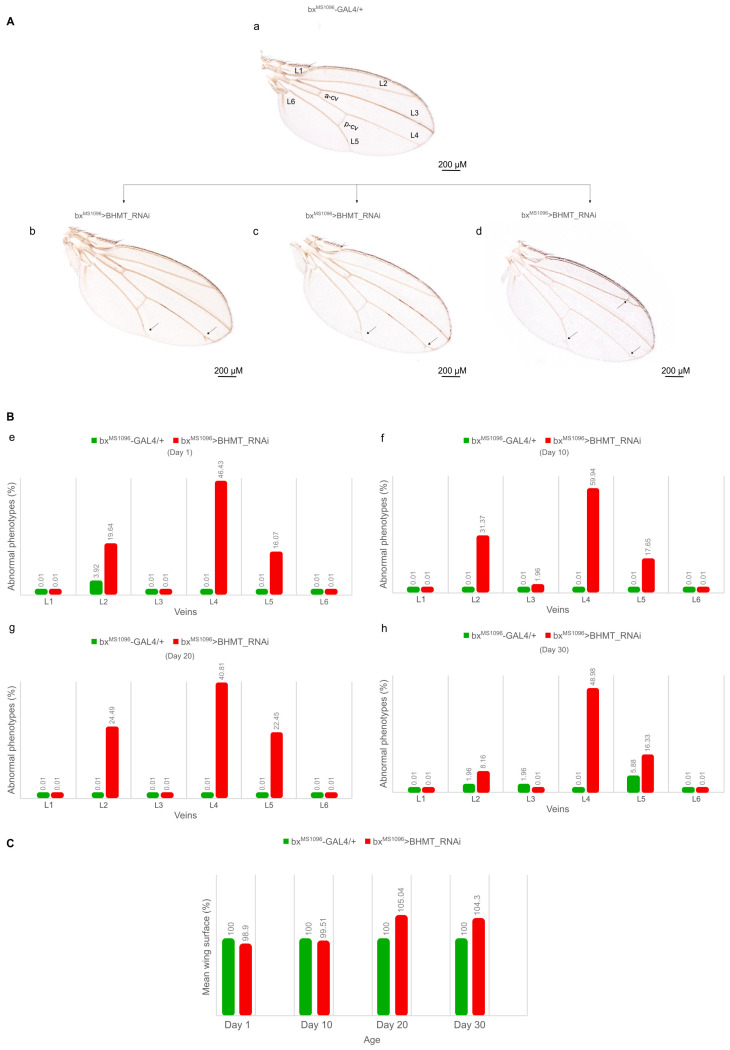
Reduced levels of *dBHMT* gene expression disrupts wing morphology and architecture in *Drosophila*: age-dependent profiles. (**A**) Wing images with differential vein pathology of male mutant flies subjected to *dBHMT* gene silencing (bx^MS1096^>BHMT_RNAi) (**b**–**d**), as compared to control fly populations (bx^MS1096^-GAL4/+) (**a**). Naming of normal (control) wing veins is indicated (L1–L6, a-cv and p-cv). Scale bars: 200 µm. (**B**) Bar charts presenting abnormal vein phenotypes of male transgenic flies (bx^MS1096^>BHMT_RNAi), as compared to control settings (bx^MS1096^-GAL4/+), during aging. (**C**) Quantification of wing areas of *dBHMT* mutant (bx^MS1096^>BHMT_RNAi) and control (bx^MS1096^-GAL4/+) flies during aging. Manually dissected wings from at least 50 male flies of each genotype were thoroughly examined and carefully quantified.

Regarding wing morphogenesis, transgenic flies with reduced dBHMT protein contents, specifically in the wing disc (bx^MS1096^>BHMT_RNAi), were collected on days 1, 10, 20 and 30 of life, and (adult) wing structural architecture was examined via a stereo-microscopic approach. The obtained phenotypes seem to vary, with abnormal vein formation being the most frequent pathogenic feature detected (Figure 12A(b–d)) compared to normally developed (typical) fly wings (bx^MS1096^-GAL4/+) (Figure 12A(a)). The most striking morphogenetic defects were identified in the L4 wing vein, followed by architectural pathologies in the L2 and L5 veins during aging (1–30 days post-birth) compared to control populations (Figure 12B(e–h)). Of note, compared to control settings, *dBHMT*-targeted flies, specifically in the wing disc, are characterized by a slightly smaller average wing surface on days 1 and 10 of life, with the average wing surface area being detectably increased (~5%) at the older adult fly ages of 20 and 30 days (Figure 12C). Given the relatively mild pathology developed, the dBHMT-mediated conversion of betaine (and homocysteine) to methionine does not seem to play a major role in both eye and wing formation or structural integrity during *Drosophila* aging.

## 3. Discussion

Nutrients are directly involved in the regulation of lifespan, aging and metabolic health of organisms, with most metabolic-related cellular activities being mediated by the mTORC1 complex, which functions as the central regulator of cell growth and metabolism in response to changes in nutrient signals, such as specific amino acid absence or (excessive) presence [19]. The essential amino acid methionine is presented as a key metabolite in many aspects of animal physiology, with its metabolism being regulated in an age-dependent manner [20]. Methionine’s primary metabolite, SAM, serves as a universal methyl donor required for most methylation reactions, including those of DNA and histones [21,22,23]. The mTORC1 signaling pathway senses the cellular levels of methionine in an indirect fashion through the SAM-specific sensor SAMTOR [7]. The SAMTOR protein is a recently described cytoplasmic amino acid sensor able to negatively regulate mTORC1 signaling by inducing its (mTORC1) dissociation from the lysosomal membrane. When SAM binds to SAMTOR, the SAMTOR-containing complex can be disrupted, and inhibition of mTORC1 signaling activity can be abolished [7] (Figure S2). SAMTOR is highly conserved among metazoans (Figure 1), and in *Drosophila* S2R+ cells lacking dSAMTOR, the dTOR pathway is resistant to methionine starvation [7], indicating that dSAMTOR plays a similar regulatory role in dTORC1 signaling to SAMTOR in human cells. 

Hitherto, the in vivo role of the dSAMTOR protein in flies’ systemic pathophysiology has not been investigated. Therefore, to reveal the importance of tissue-specific dSAMTOR loss to *Drosophila* health, lifespan and well-being, we have genetically targeted the *dSAMTOR* cognate gene in all major organ tissues through employment of the appropriate GAL4 drivers and two UAS-SAMTOR_RNAi strains. Strong *dSAMTOR* downregulation resulted in embryonic lethality in the majority of *dSAMTOR*-targeted tissues, suggesting that threshold levels of dSAMTOR activity are essential for diverse cell type functionalities, and especially for normal neuromuscular development and performance. Interestingly, moderate downregulation of the *dSAMTOR* gene in the neuromuscular system is not detrimental and is characterized by distinct phenotypes of sex- and age-dependent pathologies, with a predominantly negative impact on *Drosophila* health and lifespan. Disruption of the SAMTOR-containing complex leads to the inactivation of GATOR1 and subsequent activation of mTORC1 and, thus, suppression of autophagy [7]. It may also be the accumulation of SAM metabolite, presumably induced by the lack of the SAM-binding partner SAMTOR, that likely contributes to dSAMTOR loss-derived pathology in flies’ tissue systems. The activation of dTORC1 in *dSAMTOR*-targeted environments is strongly supported by the increased (target phosphorylation) activities of (d)p70S6K (a bona fide dTORC1 substrate) and dTOR kinases herein identified through our active kinome-wide profiling in fly brains (Figure 5). The mTORC1 complex acts as a central regulator of diverse cellular functions committed to promoting anabolism and cell growth, and its aberrant over-activation is often detected in up to 80% of human cancers [24,25,26]. mTORC1 exerts neuronal-specific activities during brain development, shaping both the signaling and physical landscapes of the brain, with these activities temporally spanning every stage of brain development [5,27]. Most importantly, mTORC1 suppresses the catabolic pathway through inhibition of autophagy and lysosome biogenesis [5]. Hyperactive mTOR signaling in the brain is associated with characteristic lesions, such as focal cortical dysplasia, seizures, macrocephaly and benign brain tumors [5]. In mutant mice that are unable to suppress mTORC1 signaling and, therefore, fail to limit their energy expenditure or activate autophagy to supply tissue cytoplasm with free amino acids, perinatal lethality occurs, thus demonstrating that mTORC1 activity must be tightly controlled to maintain normal cellular homeostasis in vivo [5,28,29]. Furthermore, failure in autophagic clearance in the brain results in the progressive and permanent destruction of neurons, causing impairment in cognition and motor control, while the inhibition of autophagy has emerged as a hallmark of neurotoxic cell death [5,30,31].

Regarding the SAM metabolite, it has long been established that it serves as a universal donor of methyl groups for almost all methyl-transfer reactions in vivo to produce a methylated substrate and SAH (S-adenosyl-L-homocysteine) metabolite [22,32]. In a SAMTOR-deficient background, cellular SAM levels increase, and the elevated SAM:SAH ratio gives rise to imbalanced cellular methylation potential, resulting in an aberrant increase in the methylation load of proteins, nucleic acids and metabolites [33]. These types of aberrant methylation events seem to critically contribute to diverse cellular pathologies, with changes in the methylation patterns of histones and nucleic acids decisively determining the epigenetic states of cells that are mechanistically associated with global alterations in gene expression profiles [22,34]. Although alterations in the methylation status do not likely account for all the detrimental effects in flies’ physiology observed herein, the functional synergism of hypermethylation with the high energy levels required by dTORC1 hyperactivation (to promote anabolism and cell growth), together with the accumulation of misfolded or excessive bio-macro-molecules due to the (dTORC1-dependent) suppression of autophagy (which could supply cells with energy and building blocks for proliferation), no longer seems to be compatible with early development in a *dSAMTOR*-deficient genetic background.

The compromised health and reduced lifespan profiles, herein described for the first time, in response to tissue-specific moderate silencing of the *dSAMTOR* gene may result either from moderate dTORC1 complex activation or from moderate SAM metabolite accumulation. Interestingly, GNMT (glycine N-methyltransferase, uses SAM as a methyl donor to produce N-methyl-glycine) downregulation (via RNAi-specific targeting) or SAMS (SAM synthase) over-expression could not affect lifespan, thereby indicating that moderate increases in SAM cellular contents cannot cause baneful outcomes in flies’ longevity [35]. In contrast, (m)TORC1 suppression has been shown to extend lifespan in different organisms, including *Drosophila* [36], strongly suggesting dTORC1′s capacity to tightly regulate flies’ longevity and lifespan [37]. Of note, dietary methionine restriction has proved to inhibit (m)TORC1 and extend lifespan in both rodents and flies [13,38,39]. Most importantly, genetic or pharmacological manipulation of SAM metabolism suffices for the expansion of *Drosophila* lifespan [35,40], thus implying that SAM, but not methionine per se, is critical for tissue and organism homeostasis in vivo. The mechanism through which the suppression of mTORC1 leads to lifespan extension includes, likely among others, the induction of autophagy [19]. The functional regulation of mTORC1 is of major importance in defining the morphology of the developing brain, promoting both the building of new proteins at some synapses and the pruning of obsolete synapses through autophagy at others [5]. Hence, the different tissue-specific phenotypes herein observed following the moderate downregulation of the *dSAMTOR* gene may be attributed to distinct degrees of balance between the inhibition or activation of dTORC1 signaling in different tissue, gender and age fly settings. Taken together, the quantitative regulation of (d)SAMTOR and SAM levels in the cytoplasm may act as a key factor in lifespan regulation. Their quantitative control is likely mediated by the tissue-dependent induction or suppression of (d)TORC1 signaling activities, which presumably become more complex at the multi-tissue and organism levels.

Methionine administration and betaine-induced elevation of methionine cellular contents in *dSAMTOR*-targeted flies, specifically in their neuronal tissues, resulted in reduced viability profiles. A high proportion of dietary protein or amino acid imbalance has been shown to reduce lifespan in *Drosophila* [41]. Furthermore, methionine, when administered at high levels, acts as toxic compound and leads to decreased longevity [42]. Regarding *Drosophila*, the toxic level of methionine has proved to be 10 mM for female flies. Nevertheless, it must be the imbalance caused by the excess of methionine over other amino acids that leads to longevity reduction [38]. Remarkably, in our in vivo experimental setting, although methionine supplementation (5 mM in the food) remained far below the toxic dose (10 mM), the dSAMTOR (moderately)-deficient background seems to produce novel complexity levels for the functional interaction of methionine with other critical amino acids. This new metabolic sub-routine, among others, could cause pathogenic accumulations of unutilized amino acids due to the inability of dTORC1 to productively sense actual methionine levels, thereby resulting in severe imbalances in protein synthesis and in a detrimentally decreased lifespan. SAMTOR serves as the major mTORC1 sensor for SAM, which directly reflects the methionine reservoir in tissue cytoplasm. Therefore, in the absence, or even modest downregulation, of the (d)SAMTOR protein, SAM, and subsequently methionine, cannot be efficiently sensed and properly quantified by (d)TORC1 and its interacting components, leading to an excessive methionine- and SAM-derived disequilibrium of amino acid network metabolism that ultimately compels flies’ organic systems to collapse in an age-dependent fashion in vivo. Surprisingly, as demonstrated using (real-time) quantitative PCR, the (mean) reduction in neuronal-targeted (endogenous) *dSAMTOR* transcript levels was measured to be 23% for the modest downregulation-carrying flies (Figure S3), thereby indicating the importance of methionine-SAM-dSAMTOR integrity, with even small or moderate decreases in *dSAMTOR* gene expression strongly compromising survival and longevity rates in enriched methionine environments in vivo. The unique genetic and nutritional synergism of (moderately) reduced dSAMTOR activity and excessive methionine (or betaine) quantity unveiled herein paves the way for the development of novel and powerful nutrigenomics (nutritional genomics) platforms able to successfully treat human pathologies via combined management of both genetic and nutritional abnormal profiles.

Betaine is a methyl derivative of glycine and participates as a methyl donor in the re-synthesis of methionine by betaine-homocysteine methyl-transferase (BHMT), utilizing homocysteine as a critical substrate [43]. To further explore the role of the methionine–SAM axis in lifespan control, we performed RNAi-mediated downregulation of the *dBHMT* gene in a tissue-specific manner throughout *Drosophila* flies’ lifetime. The genetic targeting of *dBHMT* resulted in a notably reduced lifespan and tissue-specific impaired climbing activity (indicative of neuromuscular function) during aging, a (negative geotaxis) phenotype with comparatively more pronounced pathogenic features in male populations. The only exception was observed in neuronal tissue-specific targeted female flies, which presented with a moderately increased viability profile, especially at older ages. Gender-dependent differences in the nutritional requirements of reproduction in *Drosophila* have already been reported, and it is possible that dietary control is only successful in one sex, with a negative or no effect in the other sex [44,45]. Trade-off theory proposes an interplay between lifespan and reproduction in which the cost of reproduction is an important factor affecting lifespan in a gender-specific way. Likewise, methionine restriction results in different effects on lifespan between female and male flies. In particular, Lee et al. (2014) described the relationship between gender, dietary methionine, egg production and lifespan, and explained how the status of methionine affects lifespan depending on the status of other amino acids that act through mTOR signaling [38]. The blockade of methionine re-synthesis leads to reduced levels of SAM metabolite and, thus, to the inhibition of mTORC1 signaling by SAMTOR de-repression. Furthermore, methionine depletion in human cells causes SAM exhaustion and promotes alterations in histone methylation, being mechanistically associated with widespread induction of stress response pathways and cell death sub-routines [22,46]. It seems that although methionine restriction leads to longevity and metabolic health through the suppression of (m)TORC1 activity, which is directly coupled with autophagy induction and improved insulin resistance, in *dBHMT*-targeted flies, the amount of dietary methionine intake is not sufficient for cellular homeostasis and physiological methylation events. Potential compensatory adaptations are not able to maintain the essential processes involved in methionine metabolism and, therefore, a critical threshold of (d)BHMT-regulated methionine re-synthesis is necessary for the efficient control of protein synthesis (and methylome architecture), particularly during (fly) aging.

Lifespan represents an overall sum of both the positive and negative effects of different signaling pathways related to cell/tissue type, gender, age, nutritional scheme, mutational load, genetic background and reproductive state. Hence, it is difficult to precisely determine the specific role(s) of dSAMTOR-derived signaling in lifespan control. Since the association between SAM and survival/lifespan does not function in a bidirectional fashion, a threshold of SAM levels that regulates organism lifespan is strongly suggested to exist in vivo. A similar threshold may likely act for dSAMTOR as well, with the loss or strong downregulation of *dSAMTOR* gene expression resulting in embryonic lethal phenotypes for most targeted tissues (Table 1), and a modest reduction in dSAMTOR levels causing comparatively moderate pathologies. Further investigation of the precise molecular mechanisms that describe dSAMTOR’s critical involvement in lifespan control, neuromuscular performance, tissue development and organ architecture is essential and must be promptly conducted.

## 4. Materials and Methods

### 4.1. Fly Stocks and Culturing Conditions (Drosophila husbandry)

*Drosophila melanogaster* transgenic fly strains were obtained from the Bloomington *Drosophila* Stock Center (NIH P40OD018537) (Bloomington, IN, USA): y [1] v[1]; P{y[+t7.7]v[+t1.8]=TRiP.HMJ21611}attP40 (BL: 52944), y[1] v[1]; P{y[+t7.7] v[+t1.8]=TRiP.HMJ21433}attP40 (BL: 54010), y[1] v[1]; P{y[+t7.7]v[+t1.8]=TRiP.HMC02683}attP40 (BL: 43986), w[1118]; P{w[+m*]=GAL4}repo/TM3, Sb[1] (BL: 7415), w[1118]; w[*]; P{w[+mW.hs]=GawB}D42 (BL: 8816), w[*]; P{w[+mC]=GAL4-elav.L}3 (BL: 8760), y[1] w[*]; P{w[+mC]=GAL4-Mef2.R}3 (BL: 27390), w[1118]; P{w[+m*]=GAL4}repo/TM3, Sb[1] (BL: 7415), y[1] w[*]; P{w[+mC]=Act5C-GAL4}25FO1/CyO (BL: 4414), w[1118]; P{w[+mW.hs]=GawB}Bx[MS1096] (BL: 8860) and w[*]; P{w[+mC]=GAL4-ninaE.GMR}12 (BL: 1104). The *D. melanogaster* transgenic NP1-GAL4 fly strain was kindly provided by Eric H. Baehrecke (Department of Cancer Biology, University of Massachusetts Medical School, Worcester, MA 01605, USA).

All fly stocks were maintained at 25 °C and 55–65% relative humidity, in a photoperiod of 12 h light/dark, and on a laboratory standard *Drosophila* medium (6.4% rice flour, 5% tomato paste, 3.2% sugar, 0.8% yeast, 0.8% agar, 0.4% ethanol and 0.4% propionic acid).

### 4.2. Chemicals and Reagents

Methionine (CAS 63-68-3) was provided by Sigma-Aldrich (St. Louis, MO, USA) and betaine (CAS 107-43-7) was obtained from Santa Cruz Biotechnology, Inc. (Heidelberg, Germany). The protein extraction buffer M-PER, Phosphatase Inhibitor Cocktail and Halt Protease Inhibitor Cocktail EDTA-free were purchased from Thermo Fisher Scientific (Darmstadt, Germany). The Serine/Threonine Kinase (STK) PamChip and STK reagent kit were provided by PamGene International B.V. (BJ‘s-Hertogenbosch, The Netherlands).

### 4.3. Longevity Measurement

Newly emerged (~24 h) female and male flies from each fly cross were collected and kept separate in vials (approximately 20–25 flies per vial). Flies from each vial were transferred into a fresh vial of the same medium twice a week. Survival curves were generated by counting deceased flies daily. For each viability experiment, the sample size was set to (at least) 100 flies per sex and cross (based on the literature) for statistically significant results. Methionine and betaine were employed to pharmacologically induce dTORC1 activity, and were supplemented into the fly food at concentrations of 5 and 10 mM, respectively. All viability experiments were performed at the same time for the control and RNAi-downregulated strains. Experiments were carried out three different times, using independent genetic crosses.

### 4.4. Negative Geotaxis Assay

The negative geotaxis (climbing) assay has been established as an in vivo reliable indicator for the estimation of locomotor performance in *Drosophila*. The climbing assay was performed every 10 days. Flies of both sexes were kept together. Before the experimental procedure, flies were anesthetized and divided into males and females (groups of 20–25 flies). Each experimental group was then placed in an empty 100 mL cylinder with a borderline drawn at the 60 mL mark (10 cm height). To ensure they began to climb (against gravity) simultaneously, the flies were gently tapped to the bottom of the cylinder. After a time interval of 20 sec, the number of flies that reached or exceeded the 60 mL mark was counted, and five repeats were performed for each group. The same populations were tested at different ages, excluding flies that died or flew away. The control and RNAi-targeted fly groups were examined simultaneously. The total sample size for each fly cross and gender was set to (at least) 100 flies. Experiments were performed three different times, using independent genetic crosses.

### 4.5. Statistical Analysis

The Statistical Package for Social Sciences (IBM SPSS v23.0 for Windows IBM Corp., New York, NY, USA) was used for the statistical analysis of the results. Data from longevity experiments were analyzed with the Kaplan–Meier survival test, using log rank and Breslow test statistics. Climbing graphs were drawn with an average pass rate per genotype/time point with sample standard deviation (± SSD) value. Differences between compared genotypes were evaluated through independent t-test analysis. Significance was accepted at *p* < 0.05 (*), *p* < 0.01 (**) and *p* < 0.001 (***).

### 4.6. Light Microscopy (LM)

*D. melanogaster* wings were visualized using a Nikon Digital Eclipse C1 microscope (Nikon; Tokyo, Japan). Every 10 days, for a period of 50 days, flies were anaesthetized with CO_2_, and their wings were carefully dissected and mounted with DePeX (Serva Electrophoresis GmbH, Heidelberg, Germany) onto glass microscope slides. The morphology and structure of the wings were observed separately for each gender. Wing surface area was measured using ImageJ v1.54d software.

### 4.7. Scanning Electron Microscopy (SEM)

The surface structural architecture of *D. melanogaster* compound eyes was visualized through a Phillips 515 scanning electron microscope. Female and male transgenic flies, at the age of 10, 30 and 50 days, were collected and immediately attached to aluminum stubs. Next, flies were air-dried at room temperature and coated with gold–palladium (60–40%) on a Tousimis Samsputter 2a.

### 4.8. Kinase Activity Profiling

The PamStation platform and STK (Serine/Threonine: Ser/Thr) PamChip peptide arrays, manufactured by PamGene International B.V. (‘s-Hertogenbosch, The Netherlands), were used. A typical STK PamChip contains 4 (micro-)arrays with 140 Ser/Thr peptides and 4 positive control immobilized peptides each that are covalently attached to a porous material. The peptides harbor phosphorylation sites derived from the literature or computational predictions and are correlated with one or multiple upstream kinases. Kinases in the specimens actively phosphorylate substrates on the PamChip in the presence of ATP. Phosphorylation is detected with a (phospho-)specific primary antibody, and the signal is quantified with a second FITC-conjugated antibody. Protein lysates were prepared from frozen control and RNAi-downregulated *Drosophila* heads with M-PER (cell-lysing) buffer enriched with 1:100 Phosphatase Inhibitor Cocktail and 1:100 Halt Protease Inhibitor Cocktail EDTA-free, and quantified using the Bradford assay. Next, 1 µg of total protein from each biological sample was used for Ser/Thr kinase activity profiling, according to the standard protocol provided by the manufacturer. The STK basic mix was composed of the protein lysate, 4 µL 10× PK, 0.4 µL of 100× BSA, 4.0 µL of 4 mM ATP and 0.5 µL STK antibody mix. The total volume of the STK basic mix was adjusted to 40 µL by adding distilled water. The detection mix consisted of 3 µL 10× antibody buffer (AB), 0.4 µL FITC-labeled STK antibody and 26.6 µL distilled water. After a pre-processing step of 30 cycles (blocking of peptide micro-arrays with 2% BSA), the STK basic mix was applied to each PamChip micro-array. Next, the micro-arrays were incubated and washed for 60 cycles (30–90c). Subsequently, the detection mix was added to the PamChips, and incubation with FITC-labeled STK antibody and image recording with a CCD camera (after 10, 50 and 200 msec) were operated for the next 30 cycles, and a final read was carried out after the end (cycle 124). Data normalization and visualization were performed using the BioNavigator Analysis Software Tool (BNAST).

### 4.9. RNA Extraction and RT-qPCR

Total RNA was isolated from *dSAMTOR*-targeted fly heads (manually dissected) using TRIzol™ monophasic solution of phenol and guanidine iso-thio-cyanate (Invitrogen, Thermo Fischer Scientific, Waltham, MA, USA) according to the manufacturer’s instructions. The concentration and quality of isolated RNA were determined using a NanoDrop One UV-Vis Spectro-photometer (Thermo Fischer Scientific, Waltham, MA, USA). RNA samples were reverse-transcribed into complementary DNA (cDNA) using the SuperScript™ IV First-Strand Synthesis System (Invitrogen, Thermo Fischer Scientific, Waltham, MA, USA), following the manufacturer’s protocol.

The relative expression of the *dSAMTOR* gene was investigated through reverse transcription real-time quantitative polymerase chain reaction (RT-qPCR), using *dSAMTOR*-specific oligonucleotide TaqMan™ probes (Dm01831401_s1; Thermo Fischer Scientific, Waltham, MA, USA), the TaqMan™ Universal PCR Master Mix (Applied Biosystems, Thermo Fischer Scientific, Waltham, MA, USA) and the Applied Biosystems StepOne™ real-time PCR System (Thermo Fischer Scientific, Waltham, MA, USA) according to the manufacturers’ guidelines. As an internal control for the normalization of gene-expression data, the housekeeping gene *Actin 5C* (Dm02361909_s1; Thermo Fischer Scientific, Waltham, MA, USA) was used accordingly. To ensure reproducibility, each assay was performed with technical triplicates, while three negative controls were also included in the analysis. Relative mRNA levels were calculated using the comparative 2^−ΔΔCt^ method [47], which calculates changes in gene expression as a relative fold difference between the gene of interest (*dSAMTOR*) and the reference gene (*Actin 5C*). The results are presented as a percentage of the relative gene reduction in *dSAMTOR*-targeted (specifically in neuronal tissues) flies compared to control populations.

## Figures and Tables

**Table 1:** Pathology of tissue-specific *dSAMTOR* gene downregulation, categorized into lethal and viable systemic phenotypes. S: strong downregulation; M: moderate downregulation.

Driver		dSAMTOR_RNAi^(S)^	dSAMTOR_RNAi^(M)^
	Strain		
Act5C-GAL4 (whole body)		Lethal	Viable
elav.L-GAL4 (nervous system)		Lethal	Viable
repo-GAL4 (glial cells)		Lethal	Viable
D42-GAL4 (motor neurons)		Lethal	Viable
Mef2.R-GAL4 (muscles)		Lethal	Viable
NP1-GAL4 (midgut)		Viable	Viable
ninaE.GMR-GAL4 (eyes)		Viable	Viable
bx^MS1096^-GAL4 (wings)		Viable	Viable

## Data Availability

All data are contained within the article or Supplementary Materials.

## Supplementary Materials

The following supporting information can be downloaded at: https://www.mdpi.com/article/10.3390/ijms24119676/s1.
