# Supplementary material for: Genetic Targeting of dSAMTOR, A Negative dTORC1 Regulator, during Drosophila Aging: A Tissue-Specific Pathology
Source: Int J Mol Sci. 2023 Jun 2;24(11):9676. doi: 10.3390/ijms24119676 (PMC10253371; doi:10.3390/ijms24119676)
Supplement: Supplementary file 1 [file ijms-24-09676-s001.zip › ijms-2404464-supplementary.pdf]

International Journal of
Molecular Sciences

MDPI

Article - Supplementary Materials

Genetic Targeting of dSAMTOR, a Negative dTORC1 Regulator, during Drosophila Aging: A Tissue-Specific Pathology

Stamatia A. Katarachia, Sophia P. Markaki, Athanassios D. Velentzas and Dimitrios J. Stravopodis *

Section of Cell Biology and Biophysics, Department of Biology, School of Science, National and Kapodistrian University of Athens (NKUA), 15701 Athens, Greece; skatarachia@biol.uoa.gr (S.A.K.); smarkak@biol.uoa.gr (S.P.M.); tveletz@biol.uoa.gr (A.D.V.)

* Correspondence: dstravop@biol.uoa.gr; Tel.: +30-210-727-4105

Supplementary Images

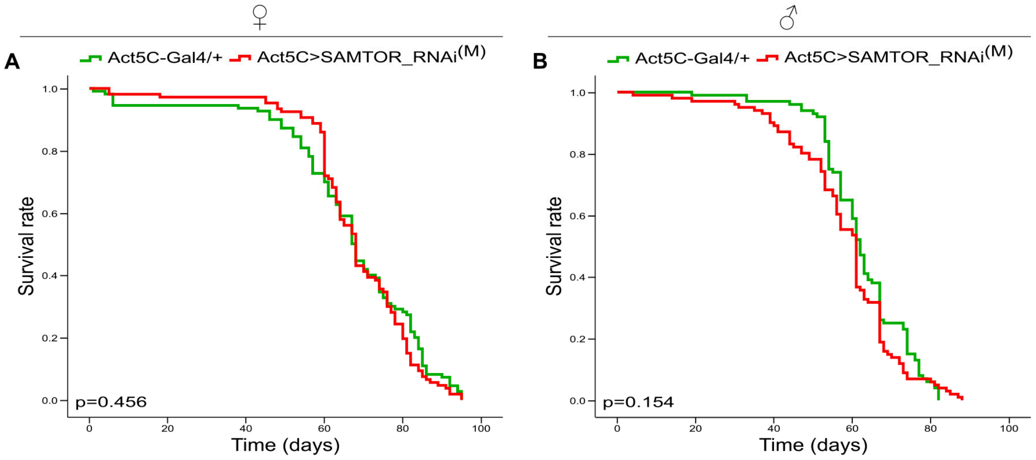

Figure S1. Moderate silencing of *dSAMTOR* gene, in all *Drosophila* tissues, does not affect life expectancy. Lifespan curves showing the survival rates of *Drosophila* transgenic female (**A**) (left panel) and male (**B**) (right panel) flies, in modest efficiency targeting of *dSAMTOR* gene expression, specifically in whole body tissues (Act5C>SAMTOR_RNAi$^{(M)}$) (red lines), as compared to control fly populations (Act5C-GAL4/+) (green lines).

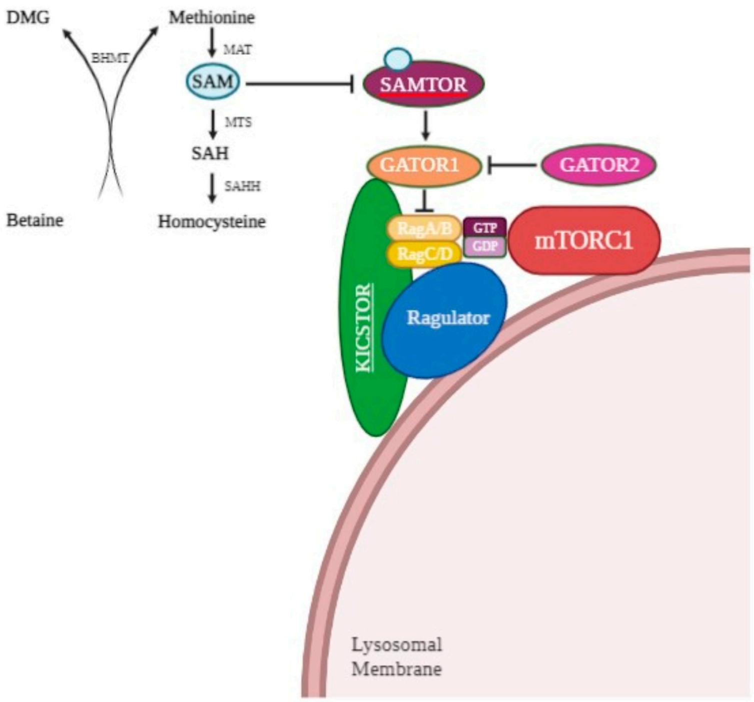

Figure S2. **Schematic illustration of lysosome-associated components being critically involved in the Methionine-sensing pathway, upstream of mTORC1**. BHMT uses Betaine to methylate Homocysteine directly into Methionine, which is, next, converted to SAM. SAMTOR has the ability to sense Methionine in the form of SAM. When SAM binds to SAMTOR, it (SAMTOR) dissociates from GATOR1 and leads to mTORC1 activation. BHMT: Betaine-Homocysteine S-Methyl-Transferase; MAT: Methionine Adenosyl-Transferase; MTS: Methyltransferase; SAHH: S-Adenosyl-L-Homocysteine Hydrolase; SAM: S-Adenosyl-Methionine.

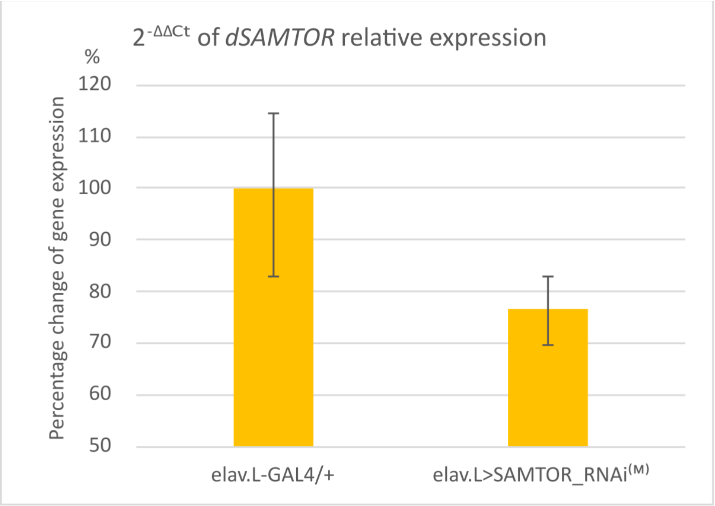

Figure S3. **Relative change in *dSAMTOR* gene expression, via RT-qPCR technology engagement**. Moderate silencing of *dSAMTOR* gene expression, specifically in fly's neuronal tissues (elav.L>SAMTOR_RNAi(M)), causes 23% (16.5% - 29.8%) relative gene-expression reduction of the gene of interest (*dSAMTOR*), compared to control fly populations (elav.L-GAL4/+).
